# Rectal Cancer: Multimodal Treatment Approach

**DOI:** 10.1155/2012/279341

**Published:** 2012-09-12

**Authors:** Manousos-Georgios Pramateftakis, Dimitrios Kanellos, Paris P. Tekkis, Nikolaos Touroutoglou, Ioannis Kanellos

**Affiliations:** ^1^Division of Surgery, Department of Surgery & Cancer, Imperial College, London SW7 2AZ, UK; ^2^Vivantes Klinikum Berlin, Berlin, Germany; ^3^Nevada Cancer Center, Las Vegas, NV, USA; ^4^Aristotle University of Thessaloniki, 541 24 Thessaloniki, Greece

Colorectal cancer is a major health problem. More than 1 million patients worldwide are diagnosed annually. It is the 3rd most common cancer type and about half a million people die of the disease each year. Incidence suggests that eating habits, lifestyle, and environmental parameters, beyond genetic background, are responsible for the disease progression. The treatment of rectal cancer has changed over the last two decades as far as surgical techniques, chemotherapy, and radiotherapy are concerned. From carcinogenesis and screening to the improvement of diagnosis and from tumor staging to the multimodal treatment approach, several fields of the management of rectal cancer as an entity have been significantly developed over the last years. Effective surgery, neoadjuvant radiotherapy, and modern cytotoxic chemotherapy have improved survival rates [1, 2].

The improvement of conventional diagnosis and the introduction of molecular screening have improved the chances of early cancer diagnosis. Computed tomography (CT), magnetic resonance imaging (MRI), virtual colonoscopy, endorectal ultrasound and positron emission tomography (PET) constitute significant means for the diagnosis and staging of colorectal cancer. Endorectal ultrasound demonstrates high accuracy in identifying penetration of the rectal wall, but is poor in assessing the N stage of the disease. CT is particularly useful in identifying other organs involvement but weak in distinguishing between T stages of the tumor, whereas MRI is accurate in identifying the presence or absence of the circumferential margin involvement. In the last five years preoperative staging has become more refined by advances in MRI imaging. Detailed assessment of MRI images is a very important parameter of the multidisciplinary rectal cancer meetings, due to its potential to predict the presence of tumor in the circumferential resection margin [3, 4].

One of the main milestones in the treatment of rectal cancer, which has overall resulted in the five-year survival improvement, is the multimodal therapy approach. The multimodal therapy of the rectal disease imposes the close interdisciplinary cooperation between colorectal surgeons, oncologists, radiologists, and radiotherapists. Locally advanced cancer is treated with combined modality therapy that includes surgery, radiation therapy and chemotherapy. It is possible to identify two patient groups, which have significantly different prognostic outcomes in terms of local recurrence. These include tumors with good prognosis that is, T1, T2, and those with poor prognosis such as T3 and T4 stage tumors. In the case of some correctly identified Tis or T1 tumors, one can find candidates for treatment with local excision alone. In the case of T2 tumors, major radical surgery should suffice, depending on the N stage of the disease. In the case of the group with poor outcome, the T3 and T4 tumors require preoperative treatment by chemoradiotherapy followed by major surgery. Neoadjuvant therapy is widely accepted as the current standard of care in the treatment of advanced rectal cancer. However, there is considerable debate regarding the best approach to neoadjuvant therapy. Studies from the United States have largely focused on a “long course” of preoperative radiation using conventional doses of 1.8–2 Gy per fraction over 5-6 weeks, for a total dose of 45 to 50.4 Gy. The Swedish Rectal Cancer Trial was the first randomized study to show that a “short course” of preoperative radiation, 5 Gy × 5 alone, without chemotherapy, followed by immediate surgery, resulted in significant improvement in 5-year survival and a reduction in the local recurrence rate for all stages of cancer [5, 6].

Following diagnosis and staging of a rectal tumor, a decision needs to be made with regards to the optimal method of surgical treatment. A few dilemmas rise up during that decision stage: To save or not to save the sphincter complex is a common question. Is there a level below which an anastomosis should not be attempted, in fear of anastomotic failure? The ideal surgical technique for low rectal tumors remains controversial in the absence of randomized trials. Unfortunately, in a passionate effort to avoid a colostomy and to re-establish intestinal continuity, surgeons often compromise on the margins of resection, with tragic consequences for the patient (local recurrence, anastomotic failure, gastrointestinal tract dysfunction and/or pelvic pain).

The introduction of total mesorectal excision (TME) by Heald in 1981, today seen as the “gold standard” of rectal cancer surgery, reduces considerably the frequency of local recurrence and increases disease-free survival rates [7]. The type of operation that can be offered to a patient with rectal cancer depends on tumour stage and on the location of the tumour in relation to the surgical anatomy. The rectal cancer NCI consensus recommended localizing the tumour relation to the anal verge, which is defined as starting at the intersphincteric groove. Another important landmark defining the upper limit of the anal canal is the anorectal ring. From the surgeon's perspective, the top of the anorectal ring is the lower limit of a distal resection margin. A large, full-thickness cancer needs to be located high enough above the top of the anorectal ring to allow for an adequate distal margin if sphincter preservation is contemplated.

Several procedures are available to the surgeon, depending on disease stage and tumor location. A low anterior resection is performed in order to remove tumors of the middle and lower rectum. For a resection to be radical, a “5 cm rule” distal free margin below the tumor is important. In case of a very low anterior resection, the anastomosis is performed at the level of the dentate line either transanally, or by the use of a circular stapler.

The sphincter-saving procedures have significantly reduced the frequency of abdominoperineal resections of the rectum. The principle of the intersphincteric resection is based on an anatomic dissection plane between the internal and external anal sphincter. It can be performed for tumors less than 3 cm from the dentate line. Due to its complexity, strict inclusion criteria have to be followed, such as absence of distant metastases, local spread restricted to the rectal wall or the internal anal sphincter and adequate distal margin potentials. The local transanal excision of tumors also seems as an attractive therapeutic option because of the minor morbidity and the short recovery time. There are, however, significant issues with regards to long-term disease control, because of the inability of the technique to control regional disease. Nevertheless, ideal candidates for this approach can be identified in patients with low-risk tumors, smaller than 4 cm, and involving less than 40% of the lumen circumference. Furthermore, significant comorbidities not allowing a more radical resection can also be a decision parameter towards local resection [8–10].

Preoperative radiotherapy or chemoradiation has been used to downstage rectal tumors and to facilitate sphincter-saving surgery. In addition to the increased resectability of bulky rectal cancers, another benefit of neoadjuvant therapy seems to be the reduction of locoregional recurrence and the improved survival. But, when the options of sphincter-saving procedures fail, the surgeon still has the option of the abdominoperineal resection (APR). Described by Miles in 1908, the APR describes the removal of the rectum with the anal mechanism, followed by the creation of an end colostomy. Many factors influence the decision to perform an APR, such as tumor level and invasion, organ involvement, anal sphincter dysfunction, systemic diseases, body habitus, and many more. Therefore, the surgeon should make the final decision of operative technique upon completion of total mesorectal excision, being certain of the absence of macroscopic and microscopic evidence of cancer invasion in the circular and distal margin of expected resection. An inadequacy of providing uninvaded margins (inability to achieve clear margins of resection) can serve as an indication to perform APR [11].

The current special issue overviews rectal cancer as a surgical oncological problem, and looks at issues surgeons are faced with when dealing with that disease. T. C. Chua et al. review the modern approach to rectal cancer surgery at all disease time points with an emphasis on some of the controversies and the accepted standards of treatment. The multimodal approach to the surgical management of locally recurrent rectal cancer is presented in the paper by N. M. Hogan and M. R. Joyce, including details on presentation, risk factors, preoperative preparation, contraindications and resectability, and also palliation. Furthermore, the review by I. Zlobec et al. outlines three situations in which the assessment of tumor budding may have direct implications on the treatment of rectal cancer within the multimodal approach.

A very informative current state-of-the-science on neoadjuvant and adjuvant therapy for patients with locally advanced rectal adenocarcinomas is written by J. T. Yorio et al., describing in detail the benefits of chemotherapy, radiotherapy and combined modality therapy regimens. A review by D. D. Kim and C. Eng explores the effects and outcome of the use of targeted agents in locally advanced and metastatic colorectal cancer, and patient benefits particularly in rectal cancer. Jabbour et al. retrospectively compared two groups of patients following neoadjuvant intensity-modulated radiation therapy or 3D-conformal radiation therapy for rectal cancer, and concluded that IMRT can reduce treatment breaks, hospitalization, and higher-grade toxicities compared to 3D CRT. Furthermore, the paper by J. A. Smith et al. assesses the differences in clinical, radiologic, and pathologic outcomes between neoadjuvant treatment of stage II-III rectal adenocarcinoma with conventional external beam radiotherapy or intensity-modulated radiotherapy versus high-dose-rate endorectal brachytherapy.

Technical details have also been issued by authors. A. Carrara et al. address the issue of local excision as appropriate treatment for early stage rectal cancer analyzing the risk factors for lymph nodal involvement. M. G. Pramateftakis et al. look at one of the operative parameters during abdominoperineal resection for low rectal cancer, namely, the localization of the pelvic drain. One of the technically demanding options for treating low rectal cancer by keeping part of the sphincter mechanism is the intersphincteric resection technique, which is analyzed in the paper by C. P. Spanos. Furthermore, a novel technique offering multiple advantages compared to the original TEM for rectal adenomas or early carcinomas is described by A. Carrara et al., namely, the glove port technique. Finally, G. Tsoulfas et al. address the issue of hepatic metastatic disease and the dilemma of which treatment step should come first, rectal resection or liver metastatic resection.

In order to be successful in treating rectal cancer, good oncologic outcome is the first priority. Equally important is the achievement of an acceptable quality of life for the patient. Despite advances in surgical technique along with improvements in neoadjuvant and adjuvant therapy, the surgical treatment of rectal cancer involving the pelvic floor and sphincter complex remains complicated. Patients with low rectal cancer pose difficulties with regards to optimal management and targeted strategies are needed to improve outcome in this complex cancer. Careful patient selection, high quality preoperative imaging, and functional assessment, with emphasis on sound operative technique and coordinated involvement of medical and radiation oncology should lead to superior results.

*Manousos-Georgios Pramateftakis* *Manousos-Georgios Pramateftakis* *Dimitrios Kanellos* *Dimitrios Kanellos* *Paris P. Tekkis* *Paris P. Tekkis* *Nikolaos Touroutoglou* *Nikolaos Touroutoglou* *Ioannis Kanellos* *Ioannis Kanellos*
